# Utilization of contraception among sexually active HIV positive women attending art clinic in University of Gondar Hospital: a hospital based cross-sectional study

**DOI:** 10.1186/s12905-016-0348-9

**Published:** 2016-10-21

**Authors:** Mulugeta Dile Worke, Lealem Meseret Bezabih, Mulat Adefris Woldetasdik

**Affiliations:** 1 Department of Midwifery, College of Health Sciences, Debre Tabor University, Debre Tabor, Ethiopia; 2 Department of Gynecology and Obstetrics, College of Health Sciences, Debre Tabor University, Debre Tabor, Ethiopia; 3 Department of Gynecology and Obstetrics, College of Medicine and Health Sciences, University of Gondar, Gondar, Ethiopia

**Keywords:** Utilization, Associated factors, Contraception, Human immune virus positive women, Ethiopia

## Abstract

**Background:**

Contraception helps to prevent unplanned pregnancies among human immune virus positive women. The contraceptive utilization status and associated factors were not well addressed in the study area. Therefore, this study aimed to assess utilization of contraceptives and associated factors among human immune virus positive reproductive age group women appearing at anti-retroviral therapy clinic at the University of Gondar Hospital, North West Ethiopia.

**Method:**

An institution based cross-sectional study was conducted among 397 systematically selected HIV positive reproductive age women who visited ART unit of the University of Gondar teaching referral hospital from January 8-20, 2014. The data were collected using pre tested and structured questionnaires through face-to-face interviews. The data were entered into Epi-Info version 3.5, and cleaned and analyzed using SPSS version 20. Descriptive summary of the data and logistic regression were used to identify possible predictors using odds ratio with 95 % confidence interval and *P*-value of 0.05.

**Results:**

The study revealed that the overall utilization of any type of contraception was 50 %. Of them, 4.1 % got contraception from anti-retroviral therapy unit. Fear of side effects was the most common (42 %) reason for not using contraception. Women who attended secondary education, married and who had 4-6 children were more likely to use contraception than their counterparts were; (AOR: 5.63; 95 % CI: 1.74–18.21), (AOR: 8.07; 95 % CI: 3.10–20.99) and (AOR: 3.61; 95 % CI: 1.16–11.26) respectively. However, Women between 35–49 years, had no intention to have another child and discordant couples were 83 %, 76 % and 65 % less likely to use contraception respectively than their counterparts.

**Conclusions:**

The results of this study revealed that the utilization of contraception was low. Women between 35–49 years, those who had no intention to have another child and whose partner was HIV sero-negative and fear of side effect of the contraception played an important role for not using contraception. Therefore, there is a need to give attention about integration of family planning service with HIV care and support service.

## Background

In spite of great progress over the last several decades, more than 120 million women worldwide want to prevent unintended pregnancy [1]. However, they and their partners are not using contraception. The reasons were the unavailability of services and supplies, limited choices and fear of social disapproval or partner’s opposition. In addition, factors such as, doubts about side effects, health concerns and lack of knowledge about contraceptive options and their use played the greatest role [1]. On the other hand, millions who are using Family Planning (FP) to avoid unintended pregnancy fail for a variety of reasons [2]. The reasons are, lack of clear instructions on how to use the methods properly, inability to get a method best suited to them, improper preparation for side effects and supplies ran out [2].

Unintended pregnancy is a common problem in both HIV positive and HIV negative women. Though it is the problem of the whole world, Sub-Saharan Africa is the home of 60 % of HIV positive people and half of this population group is females [3]. An analysis of focus countries in the president’s emergency plan for Acquired Immune Deficiency Syndrome (AIDS) relief indicated that contraception lowers the number of infants with HIV by 178 each year in Guyana to 120,256 annually in South Africa [4]. In addition, in studies conducted in Kenya and Malawi, of HIV-infected women, even though nearly three-quarters did not want more children either within the next 2 years or ever, only 32 % in Kenya [5] and 20 % in Malawi [2, 5] were using contraception.

In a study conducted among sexually active people living with HIV/AIDS seeking services, the utilization of FP methods was 87.3 % [6]. Among the types of FP methods used by respondents, condoms and modern FP methods other than condom were mentioned by 43.4 % and 36.9 % of the respondents respectively [6].

According to family health international report in 2013, there are over 100,000 pregnancies of HIV positive women and over 12,000 HIV positive births annually in Ethiopia [7]. MTCT is the predominant mode of transmission in children under 15 years of age [7]. However, studies conducted in UGH and Addis Ababa HIV/AIDS care center, utilization of family planning by HIV positive women was ranged from 34.2 % to 43.2 % [8, 9]. Dual method of contraception practiced by 31 % of the women while 27.4 % used condom alone. Sterilization was used only by 1.2 % of sexually active women while 8.7 % used only traditional methods [10].

On the contrary, studies conducted in Tigray zonal hospitals, Kola Diba and Asela hospital for HIV positive women, family planning users ranged from 46.3 to 76.5 % [11–13]. The methods used were condom, abstinence, injectable, pills, and implants. The reasons mentioned for choosing the methods were; health professional advice and observed friend experience [11]. Among the condom users, 87.6 % used condom always while 12.31 % only sometimes. The reason mentioned for condom use includes 52.3 % advised by health professionals, 90.3 % to prevent pregnancy, 74.36 % to prevent cross transmission, while for 17.4 % because their partner were negative [11]. Whereas, in Tigray Hospitals, the most commonly used methods, 59.9 % was dual contraceptives, out them 92.4 % reported utilizing condom consistently [12]. However, sexually active women in Asela did not use condoms [11]. The most common reason for the method choice was health professional advice as responded with 56 % of the respondents. Out of those who were not using family planning method, 42.9 % expressed their desire to use family planning in the future [13]. This shows that Ethiopian women have not been very successful in achieving their reproductive intentions.

In settings where HIV prevalence is high, management of sexual and reproductive health of HIV-infected women is critical to reduce HIV transmission and maternal mortality. However, family planning utilization and factors associated with it have not been well understood in resource limiting settings like Ethiopia.

Therefore, the main aim of this study was to describe family planning utilization which will help in estimating the family planning needs of HIV positive women and which in turn could help to prepare the necessary resources and flourish programs for better reproductive health services. The other main purpose of the current study was addressing the knowledge gap with regard to factors associated with family planning utilization among HIV positive women. Understanding the factors benefit in a way that patients as well as care givers intervene on those factors. This study is also believed to benefit many concerned stakeholders in decision making and policy development.

## Methods

### Setting

This institution based cross sectional study was conducted in an ART clinic of the University of Gondar teaching referral Hospital between the 8^th^ and 20^th^ of July 2014. University of Gondar Hospital is one of the oldest academic institutions in Ethiopia. It has produced a number of health professionals since more than half a century ago. The University situated at the heart of Gondar city found in Amhara Region, North West part of Ethiopia, which is located at 727 kilometers away from Addis Ababa (the capital city of Ethiopia). The hospital provides different inpatient and outpatient services to the population in the surrounding area of Gondar town and the nearby zones. In GUH ART unit, until January 30/2014, around 11081 (10122 adults and 959 pediatric) were registered in chronic care follow-up. Out of this 7554 (6910 adults and 644 pediatric) were on ART. From registration book at family planning unit of GUH, 1924 women (excluding the repeat ones) started different methods of contraceptive methods from February 1/2013 to January 30/2014. The most used methods as a contraception were 872 (45 %) condoms, 445 (23 %) pills and 343 (18 %) deoxy medroxy progesterone acetate.

### Participants

#### Source population

- The source population was all people living with HIV/AIDS who attending the University of Gondar Hospital, ART clinic,

### Study population

- Systematically selected sexually active HIV positive women between 18 and 49 years old getting services from GUH, ART clinic.

### Exclusion criteria

- Sexually active clients, registered and receiving services in GUH for less than six months
- People living with HIV/AIDS who were unable to communicate were excluded.

### Sampling technique and procedure

A sample size of 397 was Determined using single population proportion formula.

$$ \mathrm{n} = \frac{{\mathrm{Z}}^2\mathrm{p}\left(\mathrm{p}\ \hbox{-}\ 1\right)}{{\mathrm{d}}^2} $$

with the following assumptions: proportion (P) of a population practicing family planning to be 34 % as estimated from the study conducted in Tigray, Ethiopia among HIV infected women [12], a confidence level (CI) of 95 %, and marginal error (d), 5 % and 15 % non-response rate.

The University of Gondar teaching referral hospital was selected purposively. A total of 11081 people who was registered in chronic care follow-up of ART unit who lived at least six months prior to the study period were enumerated. Finally, systematic selection was done to identify study subjects.

### Variables

The dependent variable was Utilization of contraceptives, and the independent variables were demographic, socioeconomic characteristics, information on contraceptive utilization, and associated factors.

### Operational definitions

Active client: A client who received HIV/AIDS care services from the GUH, ART clinic at least once in the last six (6) months.

Sexually Active: A client who had sexual intercourse at least once in the last 3 months.

Family planning utilization: This refers to use of any form of either modern or traditional family planning (FP) method in the last one month.

Current use of FP method: Respondents who responded positively after being asked whether they are currently doing anything to delay or avoid pregnancy.

Modern FP methods: This refers to family planning methods such as pills, injectable (Depo-Provera), condoms, implants, Intra uterine contraceptive devices, vasectomy, bilateral tubal ligation.

Traditional FP methods: Other family planning methods such as Lactational Amenorrhea and Fertility Awareness Based methods.

Fertility Awareness Based methods: Based on knowledge about safe and unsafe days of conception. They include methods such as changes in basal body temperature, “thickness” of cervical mucus, use of moon beads and withdrawal method.

Side effects: Symptoms attributed to the contraceptive method and generally do not require or require medical intervention for safety (counselling & reassurance suffice), to ensure client satisfaction or safety.

### Data collection

Data was collected by two nurse interviewers using a pretested structured questionnaire at working hours’. A medical record review also conducted to confirm HAART history and to obtain clinical data (WHO stage of the disease and CD4 cell count). The questionnaire was translated to local language, Amharic by experts in both languages and was translated back to English by another person to ensure consistency and accuracy. The data collection process was closely supervised by two health officers and the principal investigator. The data collectors and supervisors were recruited based on previous experience on data collection and fluency in the local language. In addition, training was given for two consecutive days on how to interview, handling ethical issues and maintaining confidentiality and privacy. The pre-test study covered 20 reproductive age group women who are taking ART in Felegehiwot hospital, which become out of the main study two weeks before the commencement of the main data collection.

Pre-test was conducted to familiarize enumerators with the administration of the interview process and for ensuring consistency. Debriefing sessions were held with the pre-test field staff and the questionnaires were modified based on lessons drawn from the pre-test. Completed questionnaire crosschecked daily for inconsistencies and completeness.

### Data analysis

Data was first checked manually for completeness and then coded, entered and cleaned by EPI-Info 3.5.3 statistical software. Then the data were exported to SPSS windows version 20 for data checking, cleaning and logistic regression. Cleaning was done by calculating frequencies and sorting. Bivariate analysis between dependent and independent variables was performed using binary logistic regression. *P* < 0.25 was used as criteria to select candidate variables for multivariate analysis. Multivariable logistic regression analysis was done to adjust for possible confounding variables. *P*-value < 0.05 with 95 % confidence interval (CI) for OR (odds ratio) was used in judging the significance of the associations. Results were presented in text, tables and charts.

### Ethical consideration

Ethical clearance and approval was obtained from the Ethical review committee of the College of Medicine and Health Science, University of Gondar. In addition, the official letter of cooperation granted by the administrative offices of the hospital. The purpose of the study was explained to the study participants and recorded verbal informed consent was secured before data collection was started and confidentiality of the information was ensured by coding. Participation was on a voluntary basis after informed verbal consent, and responses were kept confidential. The consent procedure was approved by the ethics committee for all. The interview was undertaken privately in separate area.

## Results

### Sociodemographic characteristics

A total of 397 subjects’ participated with a response rate of 96.97 %. The mean (± standard deviation (SD)) age of respondents was 32.09 ± 6.12 (SD) years and 213 (55 %) of the respondents were in the age group of 25 to 34 years. Three hundred and five (79.2 %) were orthodox Christian, 343 (89.1 %) were Amhara and, 115 (29.9 %) were illiterate. Two hundred and seven (53.8 %) were currently married, and 295 (76.6 %) were unemployed (Table 1).

**Table 1:** Sociodemographic characteristics of HIV positive reproductive age group women attending ART units of Gondar University Hospital, Gondar, Northern Ethiopia, 2014

Variables	Frequency	Percentage
Age		
18–24	37	9.6
25–34	213	55.3
35–49	135	35.1
Religion		
Orthodox	305	79.2
Muslim	72	18.7
Others	8	2.1
Ethnicity		
Amhara	343	89.1
Tigrie	39	10.1
Others	3	0.8
Education		
Can’t read	115	29.9
Read & write	61	15.8
Primary	100	26
Secondary	81	21
College &university	28	7.3
Marital status		
Married	207	53.8
Single	32	8.3
Widowed	54	14
Divorced	84	21.8
Separated	8	2.1
Occupation		
Government	60	15.6
Private	30	7.8
House wife	150	39
Daily laborer	53	13.8
CSW	19	4.9
Others	73	19

### Reproductive related factors

**Fig. 1:**
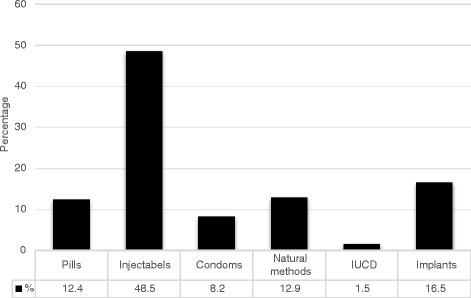
Type of contraception used by HIV positive reproductive age group women, Gondar University Hospital, Gondar, Northern Ethiopia, 2014

**Fig. 2:**
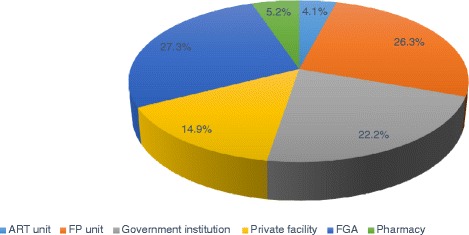
Source of contraception for HIV positive reproductive age group women in Gondar University Hospital, Gondar, Northern Ethiopia, 2014

**Fig. 3:**
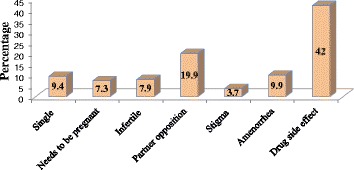
Reasons for not using contraception among HIV positive reproductive age group women in Gondar University Hospital, Gondar, Northern Ethiopia, 2014

**Fig. 4:**
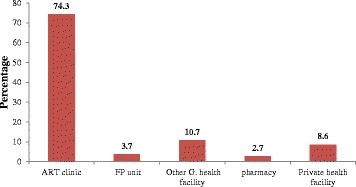
Preferred source of contraception for HIV positive reproductive age group women, in Gondar University Hospital, Gondar, Northern Ethiopia, 2014

The results of this study revealed that 60 (15.6 %) of the study subjects had at least one induced abortion. More than half, 202 (52.5 %), had 4 to 6 alive children and 281 (73 %) did not have a desire for other children. Nearly half (50.4 %) of the respondents was currently using contraception. Of this, the modern contraception utilization rate was 169 (43.89 %). The most commonly used methods, 94 (48.5 %), was injectable contraceptives (Fig. 1). Overall, 12.9 % of the respondents reported using natural methods only. Very few, 8 (4.1 %), got contraception from ART unit (Fig. 2). However, more than 83 % of respondents got counselling about contraception. Contraceptives side effects were the most common (42 %) reason for not using contraception (Fig. 3). More than 74 % preferred ART clinic to be the source of contraception (Fig. 4) (Table 2).

**Table 2:** Current utilization of contraception and other reproductive factors among HIV positive reproductive age group women attending ART units of Gondar University Hospital, Gondar, Northern Ethiopia, 2014

Variables	*N* (%)	Contraception utilization # (%)
Ever had induced abortion		
Yes	60 (15.6)	39 (65)
No	325 (84,4)	155 (47.7)
Number of alive children		
1–3	77 (20)	38 (49.4)
4–6	202 (52.5)	114 (56.4)
7–9	59 (15.3)	26 (44.1)
Intention to have another child		
Yes	104 (27)	61 (58.7)
No	281 (73)	133 (47.3)
After what time		
Soon	15 (14.4)	4 (26.7)
After two years	66 (63.5)	42 (63.6)
I don’t know	23 (22.1)	15 (65.2)
Counseling about contraception		
Yes	320 (83.1)	171 (53.4)
No	47 (12.2)	19 (40.4)
I don’t remember	18 (4.7)	4 (22.2)
How often		
On regular base	99 (30.9)	48 (28.1)
Occasionally	221 (69.1)	123 (71.9)
Current intention to use contraception		
Yes	187 (48.6)	176 (90.7)
No	198 (51.4)	18 (9.3)
Partner HIV testing		
Yes	301 (78.2)	174 (57.8)
No	13 (3.4)	5 (38.5)
I don’t know	45 (11.7)	10 (22.2)
No partner	26 (6.8)	5 (19.2)

### HIV and clinical related factors

Concerning their partner's sero-status, 301 (78.2 %) were aware of their partner's sero-status, 246 (81.7 %) seropositive and 52 (17.3 %) sero-negative. The rest, 45 (11.7 %) did not know about their partner's HIV status. The mean duration on ART is 4.8 years ± 2.4 (SD) and 92 % had disclosed their HIV status to their partner. About 97.4 % were aware of MTCT and 30 % know two or more methods to prevent it.

### Predictors of contraceptive utilization

Results of binary logistic regression showed that women who attended secondary education, house wife, government and private workers, married and those who had 4-6 live children were identified as significant predictors of Contraceptive utilization while women who had no intention to have another child, whose age were between 35 and 49 years, had no abortion, whose partner was HIV sero-negative were not associated.

In multivariable logistic regression six variables, i.e., age, marital and educational status, intention to have another child, number of live children and HIV test result of the partner were associated. When compared with those who are not able to write and read, women who attended secondary education were 5.63 times more likely to use contraception (AOR: 5.63; 95 % CI: 1.74–18.21). In addition, married women and those women who had 4-6 children were 8.07 and 3.61 times more likely to use contraceptives than their counterparts (AOR: 8.07; 95 % CI: 3.10–20.99) and (AOR: 3.61; 95 % CI: 1.16–11.26) respectively.

However, women between 35–49 years, 83 % less likely to use contraceptives than women whose age is from 18 to 24 years (AOR: 0.17; 95 % CI: 0.04–0.69). Similarly, women who had no intention to have another child and whose partner was human immune deficiency virus sero-negative were 65 % and 76 % less likely to use contraception; (AOR: 0.35; 95 % CI: 0.14–0.88) and (AOR: 0.24; 95 % CI: 0.10–0.59) respectively (Table 3).

**Table 3:** Bivariate and multivariate analyses of variables associated with contraceptive use among aged 18–49 years HIV positive women, attending ART units in Gondar University Hospital, Gondar (*N* = 385) Northern Ethiopia, 2014

Variable	Crud OR [95 % CI]	Adjusted OR [95 % CI]	*P*-value
Age in year			
18–24	1.00	1.00	0.32
25–34	1.23 [0.61, 2.48]	0.51 [0.14, 1.93]	0.01
35–49	0.47 [0.22, 0.97]	0.17 [0.04, 0.69]	
Educational status			
Can’t read	1.00	1.00	
Read & write	2.04 [1.09,3.84]	2.57 [1.08,6.12]	0.03
Primary	1.73 [1.00,2.99]	1.49 [0.49,3.32]	0.32
Secondary	3.89 [2.12,7.13]	5.63 [1.74,18.21]	0.004
College & university	1.50 [0.65,3.46]	0.89 [0.18,4.48]	0.89
Marital status			
Divorced/separated	1.00	1.00	
Married	6.36 [3.69,10.98]	8.07 [3.10,20.99]	0.00
Single/Widowed	0.76 [0.39,1.51]	1.13 [0.37,3.45]	0.82
Occupation			
Student/Others	1.00	1.00	
Government	3.31 [1.62,6.77]	1.24 [0.34,4.52]	0.73
Private	3.84 [1.56,9.44]	2.19 [0.47,10.16]	0.31
House wife	2.31 [1.29,4.13]	0.98 [0.37,2.55]	0.97
Daily laborer/CSW	1.47 [0.71,3.04]	0.91 [0.30,2.75]	0.87
Ever had abortion			
Yes	1.00	1.00	
No	0.49 [0.27,0.87]	1.09 [0.47,2.55]	0.82
Intention to have another child			
Yes	1.00	1.00	
No	0.63 [0.40,0.99]	0.35 [0.14,0.88]	0.02
Number of alive children			
None	1.00	1.00	
1–3	1.88 [0.89,3.99]	2.05 [0.59,7.02]	0.25
4–6	2.50 [1.29,4.87]	3.61 [1.16,11.26]	0.02
7–9	1.52 [0.69,3.37]	1.81 [0.50,6.52]	0.35
Partner HIV test result			
Positive	1.00	1.00	
Negative	0.31 [0.16,0.58]	0.24 [0.10,0.59]	0.00
I don’t know	0.29 [0.02,3.28]	0.18 [0.01,3.06]	0.23

## Discussion

The study revealed that overall utilization of contraception by HIV positive reproductive age group women was 50.4 %, which was consistent with study in Tigray zonal Hospital (46.3), Ethiopia [12] and higher than the studies carried out in Amhara region (33.2 %) [8], Gondar University Hospital ART clinic before six years (34.2 %) [13], Addis Ababa (39.4 %) [10] and Kenya (32 %) [14]. The high utilization in this study might be due to the fact that there were variations in time for counselling and repeated counselling, which showed there is a need to promote the practice. However, it is significantly lower than the finding from Asella hospital (57.37 %) [11] and Uganda (87.3 %) [4]. The low utilization in this study might be due to the relative low patient flow in referral hospitals, which showed there is a high patient flow in institutions lower than referral hospitals. It may be also due to shortages of the variety of family planning methods in ART units. Whereas, the difference with the Uganda study might be due to the sociodemographic differences between both countries.

In the study, factors associated with this utilization were fear of side effects, negative HIV test result of the partner, desire not to have another child, age, number of live children, educational status and marital status.

The study revealed that women who had fear side effects of contraceptive methods were 43 % less likely to utilize contraceptives than those who had no fear of side effects (OR = 0. 57: 95 % CI 0.21–1.57) which is in line with studies conducted in Tigray zonal hospitals [12] and Uganda [4]. Lower utilization of contraceptives by women who had fear of side effects of the contraceptive methods may be due to fear of the outcomes of side effects associated with contraceptives and their management. It may be also due to the absence of strategies and policies of the Ethiopian Ministry of Health regarding addressing misconceptions and side effects of contraception.

The study also found that discordant couples were 76 % less likely to use contraceptives than concordant couples (AOR: 0.24; 95 % CI: 0.10–0.59). This finding is typically different from the study conducted in Uganda [5]. The reason might be due to the presence of high partners’ opposition (19.9 %) in this study. The other possible explanation for this might be non-linked sero-negative partners to ART unit, which is resulted from less repeated counselling about contraception.

In addition, this study indicated that women who had no desire to have another child were 65 % less likely to use contraception than those who had no desired to have another child (AOR: 0.35; 95 % CI: 0.14–0.88). The lower use of contraception in women who had desire to have another child might be due to the fact that this group of women needs to space children after two years (63.5 %). In addition, this might be due to the less desire of older women to have children.

Similarly, women whose age is from 35 to 49 years were 83 % less likely to utilize contraceptive than women whose age is from 18 to 24 years (AOR: 0.17; 95 % CI: 0.04–0.69). This is in line with the finding from Tigray zonal hospitals [12]. However, contradicted with findings from EDHS; the highest users were those 30-34 years old [8]. This lesser utilization in women from 35-49 years might be due to the expectation of women to the physiological cessation of menses and fear of side effects while age increases. It might be also due to the attitude of HIV positive women towards contraceptive utilization as a result of misconception, cultural and religious barriers.

On the other hand, having 4-6 live children was statistically significant in relation to utilization of contraceptives in HIV positive women. Women having 4-6 live children were 3.6 times more likely to use contraceptives than those who had no children (AOR: 3.61; 95 % CI: 1.16–11.26), which was in line with the study conducted in Uganda [4]. However, it is different from EDHS, which indicated 35.3 % users had 1-2 children and 22.8 % of users had five or more live children [8]. This might be due to the fact that women who have no children would have higher desire to have children than those who had 4-6 alive children. This might be also due to the association of income and the number of family members.

The study showed that women attending secondary education had significant association with contraceptive use. Women who attended secondary education were 5.63 times more likely to use contraceptives than those who could not read and write (AOR: 5.63; 95 % CI: 1.74–18.21), which was in line with the findings reported from Tigray zonal hospitals [12], Northern Uganda [4], the finding from Ethiopian Demographic and Health Survey [8] and with the Ethiopian family planning guideline report [15]. This might be due to the fact that education improves communication with partner, women’s status in the community and the influence of education on women’s decision making.

Finally, this study noted that marital status had a strong association with contraceptive use. Women who married were more than 8 times more likely used contraception than those women who were divorced/separated (AOR: 8.07; 95 % CI: 3.10–20.99). This finding is in line with studies conducted in Asella hospital [15]. This might be due to the fact that married women will have high probability having regular sexual intercourse and getting pregnant than those who are widowed, divorced and never married. This might be also due to the fact that married women could have planned pregnancy than the unmarried.

However, this study does have some inherent limitations. First, the study design makes it difficult to determine the direction of causality and there is a risk of social desirability bias whereby HIV positive women may over-report their contraceptive use because of pressure from health workers and community members to practice protected sexual intercourse. In addition, this study did not include the participants’ practice of dual contraception and was not triangulated which might be difficult to get new factors, and suggested to be studied in the future. Finally, though there are wide ranges of factors which affect utilization of contraceptive methods among HIV positive women, only individual level factors were addressed in this study. Hence, considering factors from the service providers’ side and structural barriers would have been important.

## Conclusions

In conclusion, this study showed that though there is less desire to have children in the future, the utilization of contraception was only half. Age, marital status, educational status, intention to have another child, number of live children and HIV test result of the partner were important predictors of contraceptive use among HIV positive women. Therefore, integrating family planning service with HIV/AIDS care and support services and investing in women’s education could have significant impact. In addition, through prevention of unintended pregnancy, integrated services are likely to benefit maternal and child health, prevent vertical transmission, and decrease incidence of conception -related sexual transmission to discordant sexual partners. Furthermore, it is important to explore the magnitude of an unwanted pregnancy among HIV positive reproductive age group women.

## Abbreviations

AIDS: Acquired immune deficiency syndrome
ART: Anti-retroviral therapy
CDC: Centers for disease control and prevention
CPR: Contraceptive prevalence rate
DMPA: Depot medroxy-progesterone acetate
FAB: Fertility awareness based methods
FHI: Family health international
FP: Family planning
GUH: Gondar university hospital
HIV: Human immunodeficiency virus
IUD: Intra uterine devices
LAM: Lactational amenorrhea method
MOH: Ministry of Health
MTCT: Mother-to-child transmission
PLWHA: People living with HIV/AIDS
PMTCT: Prevention of mother to child transmission
STI: Sexually transmitted infections
